# Changes in gene expression associated with vitamin A metabolism in the corpora lutea and endometria during d 15 of the estrous cycle and throughout gestation in gilts

**DOI:** 10.1093/jas/skag301

**Published:** 2026-09-18

**Authors:** Matthew D Stuehr, Kyle J Hickman, Jeffrey G Wiegert, Rebecca K Poole

**Affiliations:** Department of Animal Science, Texas A&M University, College Station, TX 77843, United States; Department of Animal Science, Texas A&M University, College Station, TX 77843, United States; Department of Animal Science, Texas A&M University, College Station, TX 77843, United States; Department of Animal Science, Texas A&M University, College Station, TX 77843, United States

**Keywords:** corpus luteum, pig, pregnancy, progesterone, uterus, vitamin A

## Abstract

Retinoids (i.e. vitamin A and metabolites) have biological importance for functions involved in growth and development, immunity, and reproduction. Previous studies involving retinoids have demonstrated positive associations with increased luteal progesterone (P4) concentrations, and similarly, progesterone has been implicated in the regulation of uterine secretion of retinol-binding protein (RBP), warranting further investigation. Therefore, the aim of this study was to identify the expression of various enzymes and binding proteins involved in vitamin A metabolism in corpora lutea (CL) and endometria during d 15 of the estrous cycle and throughout various points of gestation in gilts. Sexually mature, crossbred gilts were ovariohysterectomized on d 11, 15, 60, or 90 of pregnancy, or d 15 of the estrous cycle (cyclic d 15). Plasma was collected for P4 and vitamin A analysis. Corpora luteal and endometrial tissues were analyzed for mRNAs associated with vitamin A metabolism. There were no differences in CL count, P4 concentrations, or vitamin A concentrations by cyclic or gestational day (*P *> 0.05). Corpora lutea expression of aldehyde dehydrogenase 1 family member A1 (*ALDH1A1*) and *RBP4* mRNAs were greater on d 15 of pregnancy compared cyclic d 15 gilts (*P *< 0.05). Moreover, corpora lutea mRNA expression of *ALDH1A1* (*P *= 0.0003)*, RBP2* (*P *= 0.02), and alcohol dehydrogenase 1C (*ADH1C, P *= 0.07) were greater on d 11 of pregnancy. Corpora lutea expression of lecithin retinol acyltransferase (*LRAT*) mRNA were not different (*P *> 0.05). Endometrial mRNA expression of *ALDH1A1* was greater on cyclic d 15 compared to d 15 of pregnancy (*P *= 0.002). Endometrial mRNA expression of *ALDH1A1* and *LRAT* were greater on d 60 and 90 of pregnancy compared to d 11 and 15 of pregnancy (*P *≤ 0.0001). Endometrial expression of *RBP4* mRNA was greater on d 11 and 60 compared to d 90, and greater on d 60 compared to d 15 (*P *= 0.01). Endometrial expression of *ADH1C* and *RBP2* mRNAs were not different (*P *> 0.05). These results suggest that genes encoding enzymes involved in vitamin A metabolism (*ALDH1A1, ADH1C, RBP2*, and *RBP4*) are elevated in the CL during the period of early gestation when the anti-luteolytic mechanism of maternal recognition of pregnancy is occurring. Additionally, certain genes encoding enzymes involved in vitamin A metabolism (*ALDH1A1* and *LRAT*) are elevated mid- to late-gestation in the endometrium of pregnant pigs.

## Teaser

This research advances our understanding of how vitamin A is regulated in pig reproductive tissues, revealing distinct patterns of vitamin A metabolism and transport during key stages of gestation. Improved knowledge of these biological processes may help inform future nutritional and reproductive management strategies to improve reproductive throughput and benefit the swine industry.

## Graphical abstract

**Graphical Abstract:**
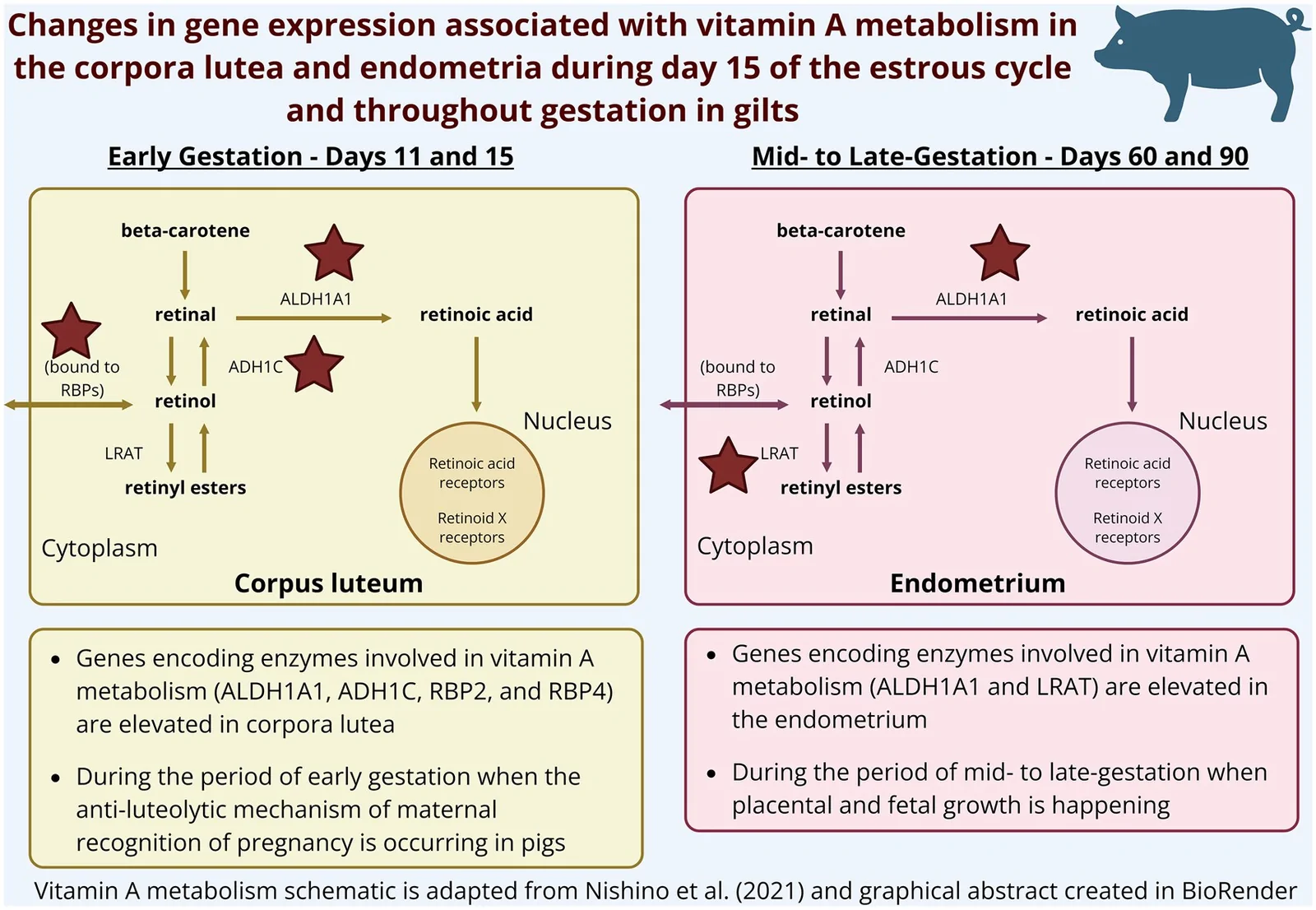

## Introduction

Proper development of corpora lutea (CL) and secretion of progesterone (P4) is crucial to the establishment and maintenance of pregnancy (McCracken et al. 1999; Christenson and Devoto 2003; Ziecik et al. 2018). Following fertilization and the initiation of embryonic development, the porcine conceptus initiates an anti-luteolytic servomechanism at approximately d 11 and 12 of gestation, which formally establishes pregnancy, and the CL maintains high P4 concentrations systemically throughout gestation (Bazer and Johnson 2014; Almeida and Dias 2022). In the swine industry, prenatal mortality (i.e. embryo and fetal mortality) makes up approximately 40% of gestational losses, decreasing litter sizes and the overall profits for pig producers (Almeida and Dias 2022). This outcome may be due to faults in the sequence of complex events involved in the development of the porcine CL and communication with other critical reproductive tissues such as the endometrium.

Retinoids have been observed to have biological importance in functions involved with cell division, growth and development, immunity, and reproduction (Gerster 1997). The terminology, vitamin A and metabolites, can encompass numerous retinoids including retinol (an alcohol), retinal (an aldehyde), and all-trans retinoic acid (Yankocy and Stenhouse 2026). Vitamin A metabolism involves various enzymatic conversions of carotenoid precursors or retinoid storage forms to intermediate and bioactive retinoid compounds. Within a cell, oxidization of retinal to retinoic acid is an irreversible reaction performed by the aldehyde dehydrogenase 1 (ALDH1) family of enzymes, with retinoic acid being considered the major bioactive component among all retinoids (Nishino et al. 2021). Conversion of retinal to retinol is a reversible reaction performed by various medium and short chain dehydrogenase/reductase family of enzymes such as retinol dehydrogenase 12 (RDH12) and alcohol dehydrogenase 1C (ADH1C, Duester 2000; Nishino et al. 2021). Following conversion of retinal to retinol, retinol is esterified to retinyl esters by enzymes such as lecithin retinol acyltransferase (LRAT), and retinyl esters are the primary storage form of retinoids in the body and are the building blocks of vitamin A metabolism (O’Byrne and Blaner 2013; Nishino et al. 2021).

In female reproduction, retinoids, predominantly isomers of retinoic acid, are involved in the proper function of various reproductive tissues such as the ovary, oviduct, and uterus, as well as in fertilization and early embryo and fetal development (Clagett-Dame and Knutson 2011; Griswold et al. 2012; Al Tanoury et al. 2013; Best et al. 2015). In the bovine CL, it has been demonstrated that concentrations of beta-carotene and retinoids naturally fluctuate throughout the estrous cycle and gestation (Chew et al. 1984; Haliloglu et al. 2002). Further, Chew et al. (1984) investigated natural retinoid concentrations in porcine plasma, follicular fluid, and CL and observed differences from those concentrations examined in cattle. *In-vitro* models have also assessed the proposed relationship between vitamin A and luteal function. Talavera and Chew (1988) examined the addition of beta-carotene, retinoic acid, or retinol to porcine luteal cell culture and observed an increase in P4 concentrations following a luteinizing hormone challenge compared to the control group, suggesting vitamin A plays a role in ovarian steroidogenesis. Additionally, exogeneous P4 has been previously shown to induce uterine secretion of retinol-binding protein (RBP) in ovariectomized gilts (Adams et al. 1981). Retinol-binding protein expression increases in the endometrium of both cyclic and pregnant gilts on d 12 and 15; however, expression of RBP decreased by d 18 in the cyclic gilts (Harney et al. 1993). These results suggest an important role for vitamin A transport in early gestation of pigs.

Because of this prior work, the present study was designed to further elucidate vitamin A’s role in CL and endometrium function during the estrous cycle and throughout gestation in pigs. The aim of this study was to identify the expression of various enzymes and binding proteins involved in vitamin A metabolism in the CL and endometrium during d 15 of the estrous cycle and throughout various points of gestation in gilts (d 11, 15, 60, and 90 of pregnancy), including: aldehyde dehydrogenase 1 family member A1 (*ALDH1A1*; enzyme responsible for the conversion of retinal to retinoic acid, Nishino et al. 2021), alcohol dehydrogenase 1C (*ADH1C*; enzyme that catalyzes the first, reversible step of vitamin A metabolism, converting retinol to retinal, Nishino et al. 2021), lecithin retinol acyltransferase (*LRAT*; enzyme that converts retinol into retinyl esters for vitamin A storage, Nishino et al. 2021), retinol-binding protein 2 (*RBP2*; binds retinol and retinaldehyde, Nishino et al. 2021), and retinol-binding protein 4 (*RBP4*; primary blood transport protein for retinol, Nishino et al. 2021). It is hypothesized that genes involved in vitamin A metabolism will have greater expression in luteal and endometrial tissue from d 15 pregnant vs. d 15 cyclic gilts. Additionally, it is hypothesized that genes involved in vitamin A metabolism will shift in expression in luteal and endometrial tissue as pregnancy advances in gilts. Overall, the goal is to improve the knowledge of vitamin A in female reproduction as well as enhance understanding of vitamin A metabolism in reproductive tissues of pigs to enhance reproductive throughput of the swine industry.

## Materials and methods

The study was completed at the Nutrition and Physiology Center at the O.D. Butler, Jr. Animal Science Complex at Texas A&M University in College Station, TX and all animal procedures followed the Texas A&M University approved IACUC 2021-0092 protocols as previously described by Hickman-Brown et al. (2025).

### Animal care and management

Sexually mature crossbred (Duroc × Landrace × Yorkshire) gilts that had experienced at least one normal estrous cycle were selected for enrollment in the study. Gilts were housed in groups in a barn with natural ventilation on solid concrete floors that were cleaned daily. Gilts were provided water *ad libitum* and 2.3 kg once daily of a mixed ration of a corn- and soybean-meal diet supplemented with standard vitamin and mineral premixes formulated to meet NRC (2012) requirements for gestation. Daily inspection of estrus was conducted and observed individuals exhibiting estrous behaviors were bred naturally to boars of proven fertility and later euthanized while under gas anesthesia via intracardiac injection of pentobarbital sodium on d 11 (*n* = 10; peri-implantation), d 15 (*n* = 11; implantation), d 60 (*n* = 6; mid-gestation), or d 90 (*n* = 6; late-gestation) of pregnancy, or were not bred and subsequently euthanized on d 15 (*n* = 5; cyclic d 15) of the estrous cycle.

### Sample collections

Following euthanasia, whole blood was collected and stored in heparinized BD Vacutainer tubes (Becton Dickinson and Company, Franklin Lakes, NJ) that were immediately placed on ice after collection. On the same day of sample collections, the whole blood was centrifuged (2,500 × *g* for 25 min at 4 °C) and plasma was separated from the sample and stored in individual 1.5 mL tubes at −20 °C until P4, vitamin A, and beta-carotene analysis. Gilts were ovariohysterectomized and both ovaries were separated from the uterus of each gilt. Following separation of the ovaries from the uterus, CL counts were performed, and tissue was frozen and later stored in −80 °C for RT-qPCR analyses. Additionally, endometrial mucosa (1 cm × 1 cm) was collected and snap frozen in a sterile 1.5 mL tube and later stored in −80 °C for RT-qPCR analyses.

### RNA extraction, cDNA synthesis, and quantitative real-time PCR analysis

Total RNA was extracted from tissues using Trizol (Invitrogen) as per manufacturer’s instructions and treated with RNase-Free DNase (Qiagen). The RNA was further purified using the RNeasy Mini Kit (Qiagen) as per manufacturer’s instructions. The RNA was quantified using a NanoDrop ND-1000 Spectrophotometer (Thermo Fisher Scientific Inc., Waltham, MA). Reverse transcription to produce complementary DNA (cDNA) was synthesized by diluting 1 μg of RNA in a 20 μL total reaction mixture before use of the SuperScript First-Strand Synthesis System (Invitrogen, Carslbad, CA) per manufacturer instructions. Negative controls without reverse transcriptase were included to verify a lack of genomic contamination. A master mix solution including 5 μL of Power SYBR Green PCR Master Mix (Applied Biosystems, Foster City, CA), 0.5 μL of both forward and reverse primers, and 3 μL of ultrapure water was used in addition to 1 μL of cDNA to make a 10 μL total reaction mix to perform RT-qPCR. Samples were then measured utilizing an ABI prism 7900HT system (Applied Biosystems, Foster City, CA). All reactions for RT-qPCR were performed at 10 μL volumes in triplicate on a 96-well plate (Life Technologies, Carlsbad, USA). Gene sequences for *ALDH1A1* (Ayuso et al. 2015), *ADH1C* (Ma et al. 2024), *LRAT* (Ayuso et al. 2015), *RBP2* (Zhou et al. 2021), and *RBP4* (Ayuso et al. 2015), used in this study were chosen from previously published work that verified expression in pig tissues (Table 1). Primer specificity for a single product was verified by its dissociation curve, and the amplification efficiency of each primer was observed to be within the range of 95% to 105%. The gene for ribosomal protein L7 (*RPL7*) was used as a reference gene value because of its witnessed stability in gilt CL and endometrial tissue across all stages in the current study and previously used in reproductive porcine tissues (Cain et al. 2024) and thus was used to normalize expression of mRNAs of interest. If quantification of gene expression was low in CL tissue (Ct *>* 30 Cycles; *ADH1C*, *LRAT*, *RBP2*, and *RBP4*), a pre-amplification method previously described by Halloran et al. (2021) was performed before conducting RT-qPCR. The relative expression for genes of interest was calculated by the ΔΔ Ct (2^−ΔΔ^) method as described in Sah et al. (2022). Outliers (values ± 3 SDs) were identified and removed from further analysis. For CL tissue, this includes four total datapoints, with one datapoint for *LRAT* (d 11 pregnant sample), one datapoint for *ADH1C* (d 11 pregnant sample), one datapoint for *RBP2* (d 15 pregnant sample), and one datapoint for *RBP4* (d 15 pregnant sample). For endometrial tissue, this includes six total datapoints, with two datapoints for *LRAT* (both d 60 pregnant samples), one datapoint for *ADH1C* (d 90 pregnant sample), one datapoint for *ALDH1A1* (d 60 pregnant sample), one datapoint for *RBP2* (d 60 pregnant sample), and one datapoint for *RBP4* (d 11 pregnant sample).

**Table 1:** Forward and reverse primer sequences of target and reference genes used for quantitative polymerase chain reaction.

Gene	Oligo name	GenBank accession no.	Primer sequence (5′–3′)	Reference
**Aldehyde dehydrogenase1 family member A1**	ALDH1A1	XM_021064911.1	F: CAGTGAAGGCCGCAAGACAAG R: TGCAGCCTCCTAAATCCATCAGA	Ayuso et al. (2015)
**Alcohol dehydrogenase 1C**	ADH1C	NM001243939	F: ACCCCCGAAGGCCTATGAAGT R: CTGGGGAACAAAGAGTGGGATGA	Ma et al. (2024)
**Lecithin retinol acyltransferase**	LRAT	NM00124492	F: CTCAAGAAGAAGGCGCTGCTCAA R: ATTATCTTCACATTCTCACAAAA	Ayuso et al. (2015)
**Retinol-binding protein 2**	RBP2	NM_214451	F: AGGAGAACCGAGGCTGGAA R: ACACCTGGTCACCGCATGT	Zhou et al. (2021)
**Retinol-binding protein 4**	RBP4	NM_214057	F: GCCGAGTGAGCAGCTTCCGAGTC R: TGCGCACACGTCCCAGTTATTT	Ayuso et al. (2015)
**Ribosomal protein L7**	RPL7	NM_001113217	F: AAGCCAAGCACTATCACAAGGAATACA R: TGCAACACCTTTCTGACCTTTGG	Cain et al. (2024)

### Progesterone, vitamin A, and beta-carotene quantification

Progesterone concentrations were measured using a double-antibody radioimmunoassay (RIA) kit (MP Biomedicals, Santa Ana, CA) per manufacturer’s instructions. The RIA was performed as previously described (Pohler et al. 2016; Reese et al. 2020; Soffa et al. 2025), and concentrations of P4 were quantified using a standard curve, which included high and low reference samples. Samples were run in a single run, and the intra-assay coefficient of variation (CV) was 6.14%. Additionally, plasma samples were shipped to Michigan State University Veterinary Diagnostic Lab for quantification of plasma vitamin A and beta-carotene concentrations using ultra-performance liquid chromatography. Plasma beta-carotene concentrations were measured in samples; however, the results were too low for quantification (<0.05 μg/mL).

### Statistical analyses

Statistical analysis comparing P4 concentrations, CL count, and vitamin A concentrations with the fixed effect for day was performed using PROC GLM in SAS 9.4. Data were subjected to the Anderson–Darling test to check normality of the distribution of data using PROC UNIVARIATE, and a *P-*value of ≥0.05 indicated that the data were not normally distributed. Normality was reassessed after exclusion of the outliers. One-way ANOVA test was performed on data with a normal distribution using PROC GLM. If data were not normally distributed, the Kruskal-Wallis test was performed with the DSCF method applied for multiple comparison analysis using PROC NPAR1WAY. Additionally, correlations between vitamin A concentrations and P4 concentrations as well as vitamin A concentrations and relative gene expression were completed using PROC CORR in SAS 9.4 with Spearman rank-order correlation performed for data that were not normally distributed and Pearson correlation for data with a normal distribution. Data is presented as mean ± standard error of mean (SEM). For all statistical analyses, the results were considered significant at *P* ≤ 0.05, trending toward significant at 0.05 < *P* ≤ 0.1, and not significant at *P* > 0.1.

## Results

### CL count, plasma concentrations of P4, and vitamin A

Comparing cyclic d 15 and pregnant d 15 gilts, no differences were observed in the CL count per animal (13 ± 2 vs. 15 ± 1, *P *= 0.57; Figure 1A), plasma progesterone concentrations (13.1 ± 5.1 vs. 22.9 ± 4.6 ng/mL, *P *= 0.15; Figure 1B), or plasma vitamin A concentrations (267.8 ± 28.1 vs. 319.0 ± 19.9 ng/mL, *P *= 0.16; Figure 1C). Additionally, when comparing across gestational d 11, 15, 60, and 90, no differences were observed in the CL count per animal (18 ± 1 vs. 15 ± 1 vs. 16 ± 1 vs. 17 ± 1, *P *= 0.42; Figure 2A), plasma progesterone concentrations (19.5 ± 4.2 vs. 22.9 ± 4.6 vs. 12.9 ± 5.0 vs. 18.2 ± 4.6 ng/mL, *P *= 0.54; Figure 2B), or plasma vitamin A concentrations (289.9 ± 18.0 vs. 319.0 ± 19.9 vs. 333.0 ± 23.3 vs. 324.8 ± 23.3 ng/mL, *P *= 0.45; Figure 2C).

**Figure 1:**
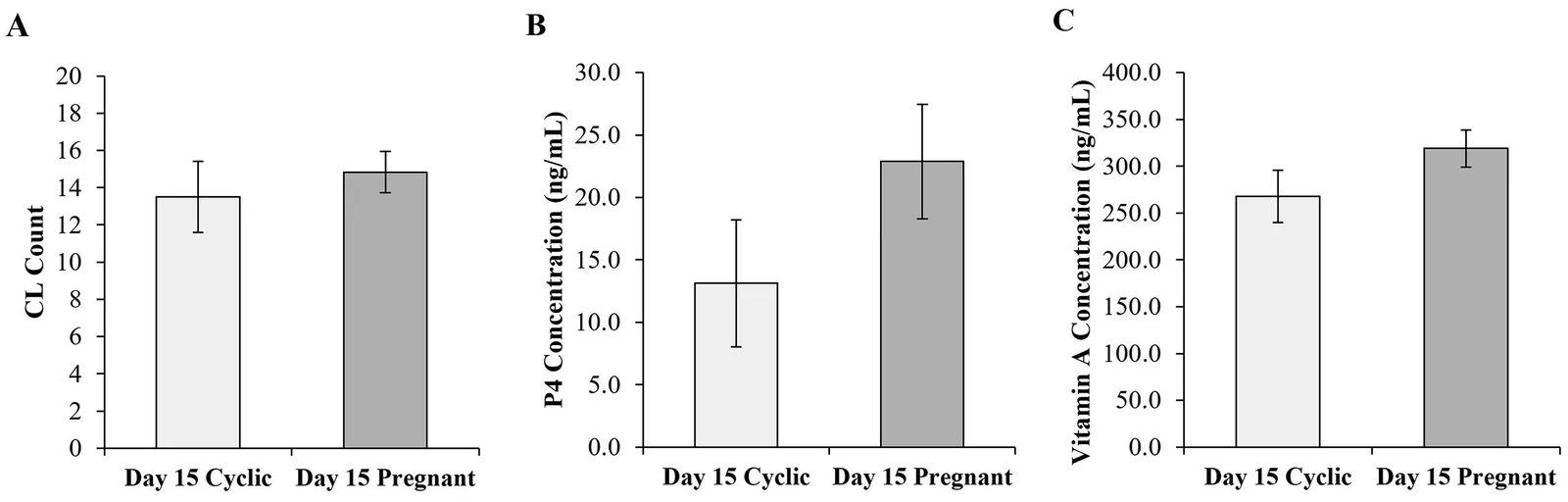
A) Corpora lutea (CL) count per animal, B) plasma progesterone (P4) concentration (ng/mL), and C) plasma vitamin A concentration (ng/mL) for gilts on d 15 of the estrous cycle (*n* = 5) or d 15 of pregnancy (*n* = 11). Data are presented as mean ± SEM.

**Figure 2:**
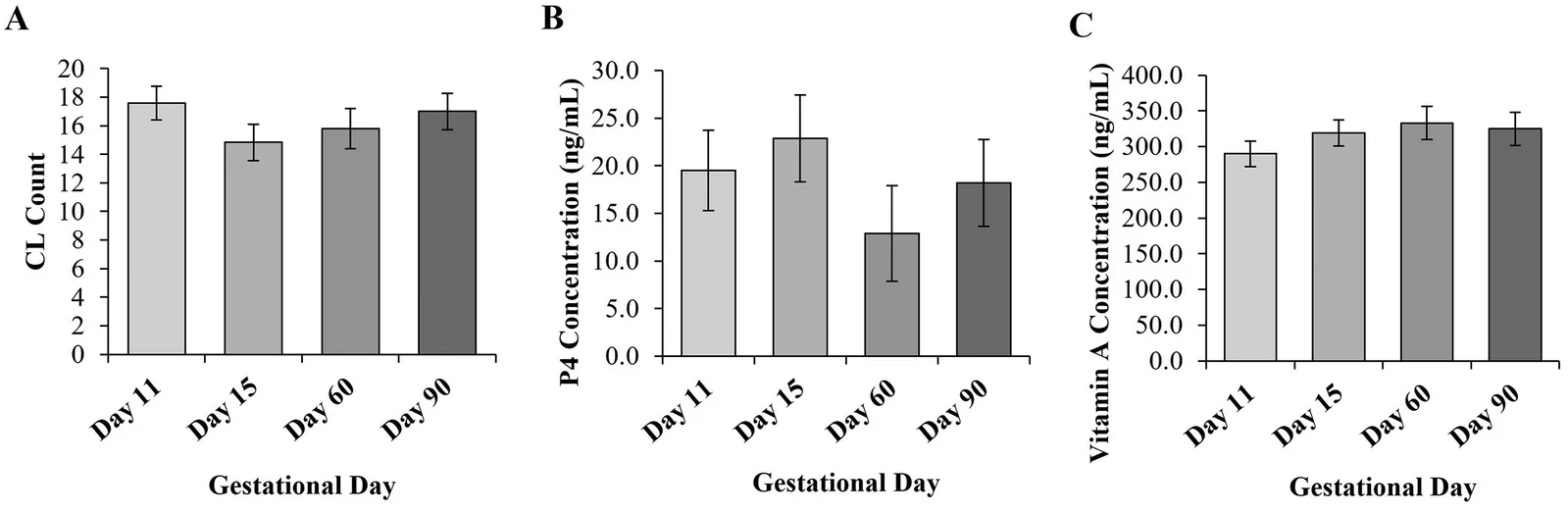
A) Corpora lutea (CL) count per animal, B) plasma progesterone (P4) concentration (ng/mL), and C) plasma vitamin A concentration (ng/mL) for gilts on d 11 (*n* = 10; peri-implantation), d 15 (*n* = 11; implantation), d 60 (*n* = 6; mid-gestation), or d 90 (*n* = 6; late-gestation) of pregnancy. Data are presented as mean ± SEM.

### Expression of mRNAs in the CL

Comparing cyclic d 15 and pregnant d 15 gilts, expression of *ALDH1A1* mRNA was greater in CL tissue from pregnant d 15 gilts (0.66 ± 0.23 vs. 1.32 ± 0.16, *P = *0.04; Figure 3A). There were no differences observed in the expression of *ADH1C* mRNA (1.13 ± 0.29 vs. 0.72 ± 0.21, *P = *0.27; Figure 3B), *LRAT* mRNA (0.94 ± 0.22 vs. 0.93 ± 0.16, *P = *0.99; Figure 3C), or *RBP2* mRNA (0.68 ± 0.20 vs. 1.02 ± 0.15, *P = *0.20; Figure 3D) between cyclic d 15 and pregnant d 15 gilts. Expression of *RBP4* mRNA was decreased in CL tissue from cyclic d 15 gilts compared to pregnant d 15 gilts (0.31 ± 0.30 vs. 1.61 ± 0.25, *P *= 0.01; Figure 3E).

**Figure 3:**
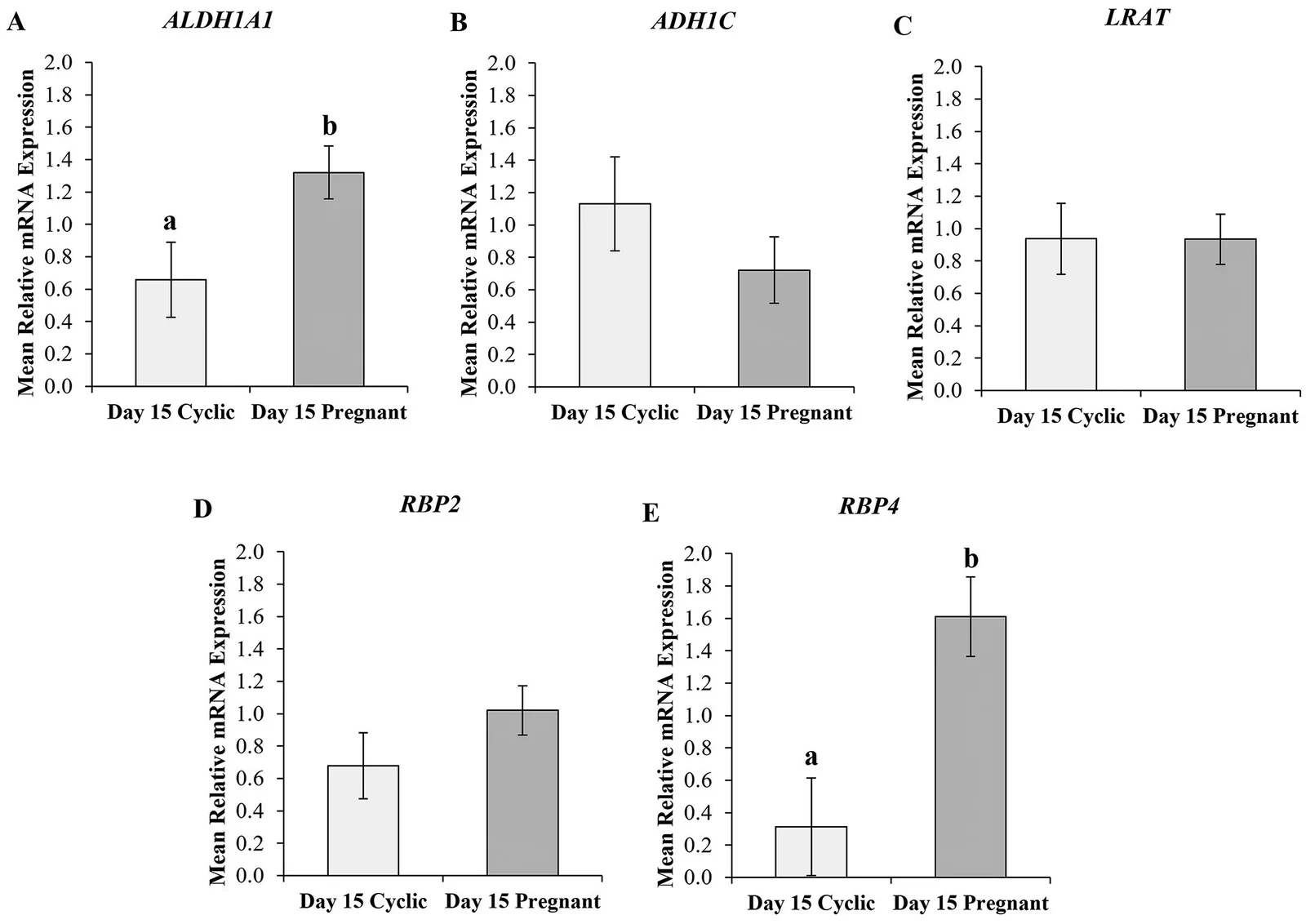
Relative mRNA expression of A) *ALDH1A1*, B) *ADH1C*, C) *LRAT*, D) *RBP2*, and E) *RBP4* of CL tissue from gilts on d 15 of the estrous cycle (*n* = 5) or d 15 of pregnancy (*n* = 11). ^a,b^Differences in superscripts indicate *P*-values ≤0.05. Data are presented as mean ± SEM.

When comparing across gestational days, expression of *ALDH1A1* mRNA was greater in CL tissue at d 11 compared to all other gestational days, and between d 15 and d 90 (2.26 ± 0.23 vs. 1.32 ± 0.16 vs. 0.85 ± 0.30 vs. 0.53 ± 0.30, *P = *0.0003; Figure 4A). In pregnant gilts, expression of *ADH1C* mRNA was greater in CL tissue on d 11 compared to d 15 (1.69 ± 0.26 vs. 0.72 ± 0.25, *P = *0.01) and tended to be greater at d 11 compared to d 90 (1.69 ± 0.26 vs. 0.96 ± 0.32, *P = *0.09; Figure 4B). There were no differences observed in the expression of *LRAT* mRNA across gestational days (*P = *0.13; Figure 4C). In pregnant gilts, expression of *RBP2* mRNA was greater in CL tissue on d 11 compared to all other gestational days (1.67 ± 0.20 vs. 1.02 ± 0.22 vs. 0.98 ± 0.26 vs. 0.65 ± 0.26, *P = *0.02; Figure 4D). There were no differences observed in the expression of *RBP4* mRNA across gestational days (*P = *0.99; Figure 4E).

**Figure 4:**
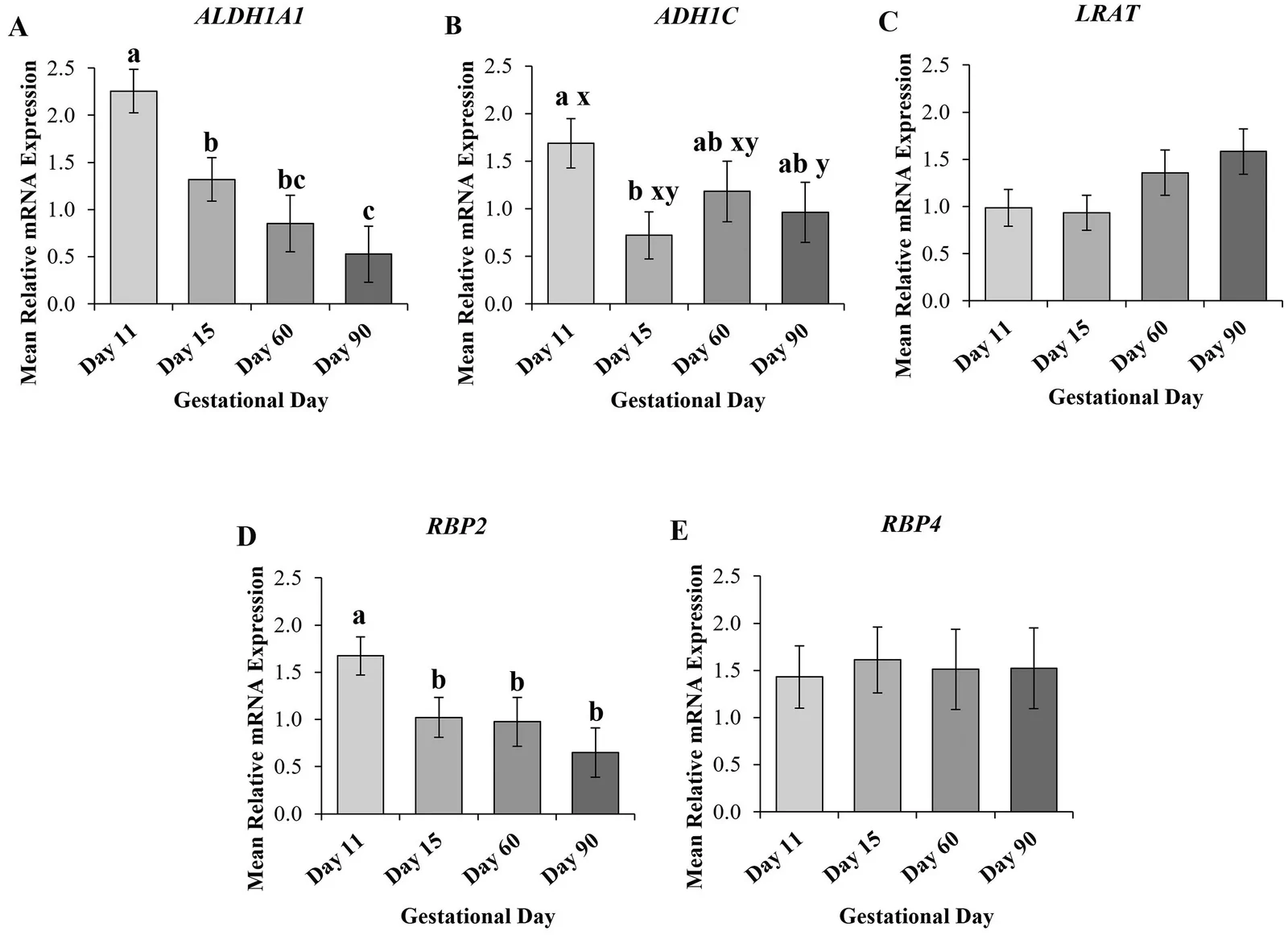
Relative mRNA expression of A) *ALDH1A1*, B) *ADH1C*, C) *LRAT*, D) *RBP2*, and E) *RBP4* of CL tissue from gilts on d 11 (*n* = 10; peri-implantation), d 15 (*n* = 11; implantation), d 60 (*n* = 6; mid-gestation), or d 90 (*n* = 6; late-gestation) of pregnancy. ^a,b,c^Differences in superscripts indicate *P*-values ≤0.05. ^x,y^Differences in superscripts indicate *P*-values >0.05 and ≤0.1. Data are presented as mean ± SEM.

### Expression of mRNAs in the endometrium

Comparing cyclic d 15 and pregnant d 15 gilts, expression of *ALDH1A1* mRNA was greater in endometrial tissue from cyclic d 15 gilts (0.63 ± 0.08 vs. 0.26 ± 0.15, *P = *0.002; Figure 5A). There were no differences observed in the expression of *ADH1C* mRNA (0.27 ± 0.24 vs. 0.61 ± 0.26, *P = *0.43; Figure 5B), *LRAT* mRNA (0.65 ± 0.21 vs. 0.51 ± 0.15, *P = *0.58; Figure 5C), *RBP2* mRNA (0.72 ± 0.62 vs. 1.56 ± 0.44, *P = *0.29; Figure 5D), or *RBP4* mRNA (0.46 ± 0.37 vs. 0.91 ± 0.26, *P = *0.34; Figure 5E) between cyclic d 15 and pregnant d 15 gilts.

**Figure 5:**
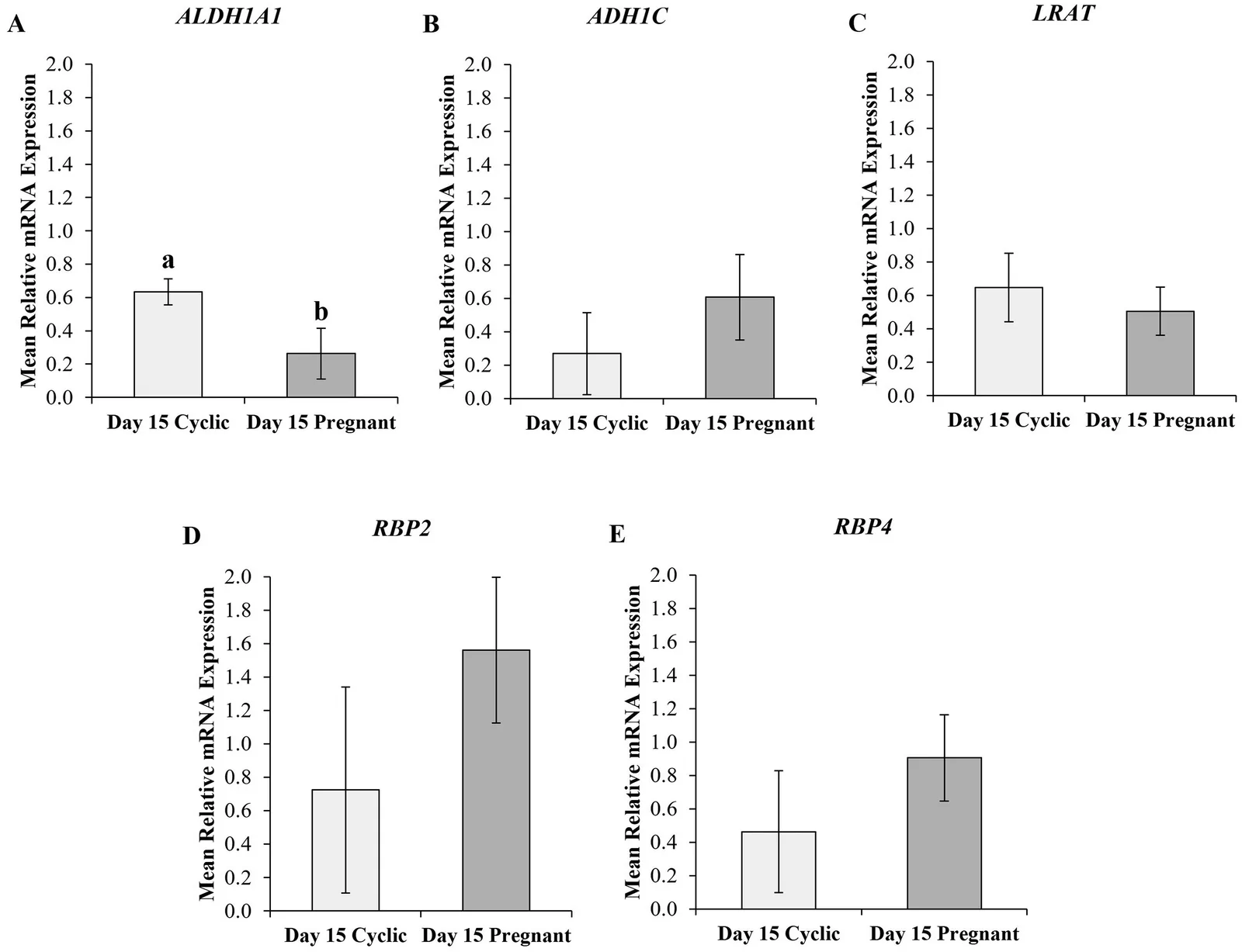
Relative mRNA expression of A) *ALDH1A1*, B) *ADH1C*, C) *LRAT*, D) *RBP2*, and E) *RBP4* of endometrial tissue from gilts on d 15 of the estrous cycle (*n* = 5) or d 15 of pregnancy (*n* = 11). ^a,b^Differences in superscripts indicate *P*-values ≤0.05. Data are presented as mean ± SEM.

When comparing across gestational days, endometrial expression of *ALDH1A1* mRNA was decreased on d 11 and 15 compared to d 60 and 90 (1.19 ± 0.50 vs. 0.26 ± 0.15 vs. 3.84 ± 0.84 vs. 4.71 ± 0.75, *P *= 0.0001; Figure 6A). There were no differences observed in the endometrial expression of *ADH1C* mRNA across gestational days (*P = *0.94; Figure 6B). Endometrial expression of *LRAT* mRNA was decreased on d 11 and 15 compared to d 60 and 90, and different between d 60 and 90 (0.55 ± 0.54 vs. 0.51 ± 0.41 vs. 14.28 ± 1.37 vs. 4.43 ± 0.79, *P *< 0.0001; Figure 6C). There were no differences observed in the endometrial expression of *RBP2* mRNA across gestational days (*P = *0.42; Figure 6D). Endometrial expression of *RBP4* mRNA was greater on d 11 and 60 compared to d 90, and greater on d 60 compared to d 15 (1.47 ± 0.26 vs. 0.91 ± 0.23 vs. 1.67 ± 0.30 vs. 0.29 ± 0.28, *P *= 0.01; Figure 6E).

**Figure 6:**
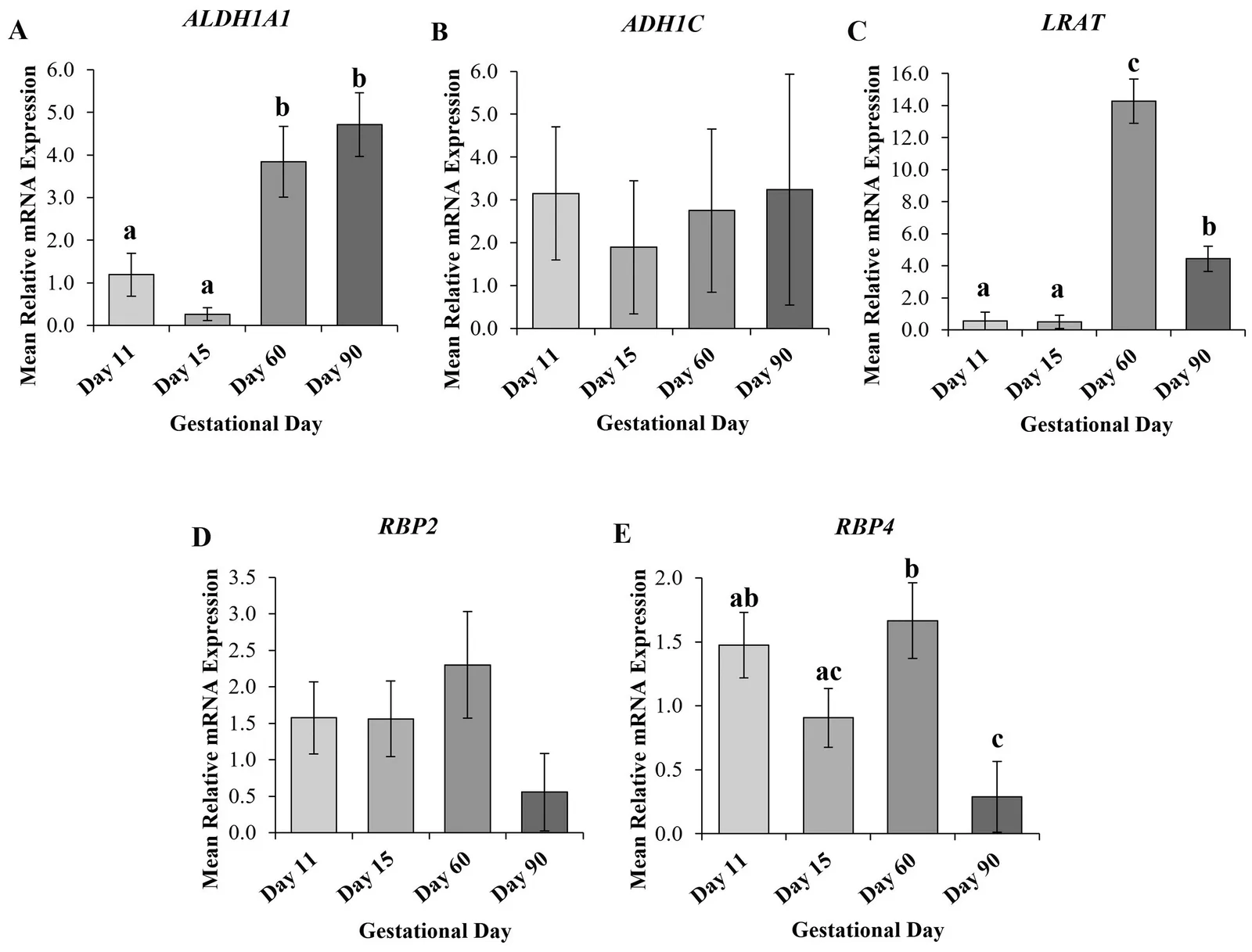
Relative mRNA expression of A) *ALDH1A1*, B) *ADH1C*, C) *LRAT*, D) *RBP2*, and E) *RBP4* of endometrial tissue from gilts on d 11 (*n* = 10; peri-implantation), d 15 (*n* = 11; implantation), d 60 (*n* = 6; mid-gestation), or d 90 (*n* = 6; late-gestation) of pregnancy. ^a,b,c^Differences in superscripts indicate *P*-values ≤0.05. Data are presented as mean ± SEM.

### Correlations with circulating vitamin A

When evaluating the correlation between circulating vitamin A and P4 concentrations, there was no relationship detected (*r *= 0.001, *P *= 0.99). As presented in Table S1, there were no significant correlations between circulating vitamin A and mRNA expression of *ALDH1A1, ADH1C, LRAT, RBP2*, or *RBP4* in CL or endometrial tissue. However, there was a tendency for a positive, weak correlation between endometrial mRNA expression of *RBP4* and circulating vitamin A (*r *= 0.299, *P *= 0.086; Figure S1, see online supplementary material for a color version of this figure).

## Discussion

Retinoids (i.e. vitamin A and metabolites) are an essential nutrient involved in numerous biological processes, including mechanisms crucial to female reproduction. It has been previously established that concentrations of beta-carotene and vitamin A fluctuate in the plasma, CL, and follicular fluid of follicles throughout the estrous cycle and pregnancy in cattle and pigs (Chew et al. 1984; Haliloglu et al. 2002). Additionally, RBP expression increases in the endometrium of both cyclic and pregnant gilts on d 12 and 15; however, expression of RBP decreased by d 18 in the cyclic gilts (Harney et al. 1993). Together, these prior findings suggest an important role for vitamin A metabolism and transport associated with swine reproduction. Because of this previous work, the present study was designed to identify the expression of various enzymes and binding proteins involved in vitamin A metabolism in CL and endometria during d 15 of the estrous cycle and throughout various points of gestation in gilts. Overall, the goal is to improve the knowledge of vitamin A in female reproduction as well as enhance understanding of vitamin A metabolism in reproductive tissues of pigs to benefit reproductive throughput of the swine industry.

During the luteal phase of the estrous cycle, following ovulation, systemic concentrations of P4 increase due to the forming CL on the ovaries, which prepare the uterus for implantation and establishment of pregnancy (Ziecik et al. 2018). In the current study, comparing both CL count and plasma P4 concentrations across groups revealed no differences, which was an expected result. The CL is especially crucial in pig pregnancy, as the pig relies solely on P4 output from the CL throughout gestation to maintain pregnancy because the placenta of pigs produce little to no P4, unlike other eutherian mammal placentas (Bazer and Johnson 2024). Similarly, plasma concentrations of P4 from the CL of pregnant pigs must be at least 4 ng/mL and must remain stable during the entirety of gestation to ensure a successful pregnancy and subsequent delivery of piglets (Ellicott and Dziuk 1973; Ziecik et al. 2018). Additionally, a study conducted by Chew et al. (1982) observed that either feeding vitamin A or beta-carotene in differing amounts or injecting beta-carotene to sham-bred pigs did not change CL numbers in early gestation compared to the control-fed group.

Vitamin A is an essential fat-soluble vitamin that is extremely important to numerous physiological processes. Vitamin A and metabolites can encompass numerous retinoids including retinol (an alcohol), retinal (an aldehyde), and all-trans retinoic acid (Yankocy and Stenhouse 2026). Several studies evaluating supplementation of vitamin A orally or through injection to pigs have been completed; however, minimal research has examined endogenous concentrations of vitamins throughout gestation or comparing maternal concentrations during pregnancy to cyclic amounts as observed in the current study. The quantification of plasma vitamin A concentrations revealed no differences in d 15 cyclic or pregnant pigs or throughout various points in gestation. However, it is important to note that gilts were group housed and thus individual feed intake was not captured in the current study. Therefore, potential differences in daily feed intake, and thus consumption of dietary vitamin A, could impact interpretation of results in the current study. In humans, pregnant women have an increased need for vitamin A during pregnancy because of the increased demand for vitamin A from the fetus, most observed in the third trimester, and also from the physiological increase of maternal blood volume that occurs during pregnancy (Sapin et al. 2000; Bastos Maia et al. 2019; Jouanne et al. 2021). While nutritional requirements of vitamin A in livestock species are not as researched as in humans, it is assumed that pregnant pigs may require more vitamin A during gestation, similar to humans (Anderson et al. 1970; Mahan and Vallet 1997). Indeed, recent research in multiparous sows shows that elevating dietary vitamins and minerals from d 70 to 110 of gestation reduces sow farrowing duration and increases the number of pigs weaned per litter (McClellan et al. 2025), suggesting the modern hyperprolific sow may have greater nutritional requirements than previously thought. Future examinations investigating nutritional requirements and circulating concentrations of vitamin A in cyclic and pregnant pigs will improve swine nutritional guidelines and the overall knowledge of pig reproduction, boosting swine production efficiency.

The measurement of beta-carotene in maternal plasma was also conducted; however, concentrations were too low for quantification. Previous research has revealed that beta-carotene concentrations in the plasma of pigs are lower than that of other mammalian species, including the cow, the horse, and the human, even after supplementation (Schweigert et al. 2001). In the same study, Schweigert et al. (2001) measured tissue concentrations of beta-carotene demonstrating low concentrations in the liver, adrenals, and CL (Green and Fascetti 2016). It is hypothesized that because pigs have white adipose tissue, they are believed to be low absorbers and accumulators of beta-carotene, unlike species with yellow adipose tissue, which are animals whose adipose tissue gains a yellow color from absorbing high beta-carotene amounts from ingested forages (Schweigert et al. 2001; Green and Fascetti 2016). Furthermore, the average conversion rate of beta-carotene in pigs is generally regarded as the lowest conversion rate among livestock species (Schweigert et al. 2001; Green and Fascetti 2016; Shastak and Pelletier 2023). Overall, the pig seems to be relatively inefficient at absorbing beta-carotene and converting it the vitamin A compared to other species (Green and Fascetti 2016).

Vitamin A metabolism is a crucial aspect to how vitamin A is transported, stored, and functions throughout the body, as each retinoid form serves a unique function following synthesis. In the current study, the mRNA expression of key enzymes and binding proteins involved in vitamin A metabolism were quantified in the CL and endometrium of d 15 cyclic or pregnant pigs throughout various points in gestation. Examining the gene, *RBP4*, coding for RBP4, which is a blood transport protein that binds retinol from the liver and delivers to peripheral tissues, there was an increase in mRNA expression in the CL on d 15 of pregnancy compared to d 15 cyclic gilts. Moreover, the gene, *RBP2*, had increased mRNA expression in the CL on d 11 of pregnancy compared to all other gestational days. The gene, *ADH1C*, which codes for the enzyme, ADH1C, that oxidizes retinol to retinal, showed increased mRNA expression in the CL on d 11 of pregnancy. Finally, for the gene, *ALDH1A1*, coding for the enzyme, ALDH1A1, which irreversibly oxidizes retinal to retinoic acid, increased expression in the CL was observed on d 15 of pregnancy compared to d 15 cyclic gilts. Additionally, increased expression of *ALDH1A1* was observed on d 11 of pregnancy compared to all other gestational days. Similar findings have also been observed for ALDH1A1 in the bovine CL during early pregnancy (Hughes et al. 2020, 2022). Therefore, elevated CL mRNA expressions of *ADH1C, ALDH1A1, RBP2*, and *RBP4* during early gestation in the pig may indicate that delivery of retinol and retinoic acid synthesis is favored during this period of gestation in the CL. Although, the current study is limited to the mRNA expression, with no protein quantification, thus warranting future studies. Furthermore, gestational d 11 is an important pregnancy day in the pig, as around this time, the porcine anti-luteolytic mechanism, which is required to protect the CL and maintain pregnancy, actively occurs (Meyer et al. 2019; Bazer and Johnson 2024). Starting between pregnancy d 11 and 12, the elongating conceptuses increase secretion of estrogen, which redirects endocrine transport of prostaglandin F2α to exocrine, whereby the CL is protected from luteolysis and the pregnancy is maintained (Meyer et al. 2019; Bazer and Johnson 2024). A second and more sustained increase of estrogen from the conceptuses is believed to occur between d 15 and 25–30 of pregnancy, which further reinforces CL protection (Meyer et al. 2019). Furthermore, conceptus expression of porcine-specific interleukin 1beta 2 (IL1B2) has suspected involvement in the maternal recognition of pregnancy mechanism in the pig as IL1B2 expression from the conceptuses between d 12–18 of gestation is required for conceptus elongation and is a prerequisite for conceptus estrogen production (Bazer and Johnson 2014, 2024; Almeida and Dias 2022). Taken together, future investigations should examine the actions of these binding proteins and enzymes and/or retinoic acid in conceptus elongation and the anti-luteolytic maternal recognition of pregnancy signaling mechanism in pigs.

In the endometrium, mRNA expression of key enzymes and binding proteins involved in vitamin A metabolism were elevated in mid- to late-gestation. For the gene *ALDH1A1*, there was significantly increased expression in the endometrium observed on d 60 and 90 of pregnancy compared to all other gestational days. Furthermore, examining the gene *LRAT*, which codes for LRAT, an enzyme that converts retinol into retinyl esters for vitamin A storage, there was significantly increased expression in the endometrium observed on d 60 and 90 of pregnancy compared to all other gestational days, with the greatest expression observed on d 60. Interestingly, mRNA expression of *RBP4* was greater on d 11 and 60 compared to d 90. Although, similar to the CL tissue, the current study is limited to the mRNA expression in the endometrium, with no protein quantification, thus warranting future studies. Based on the results, the specific increase of RBP4 in the endometrium on d 11 of pregnancy align with previous studies in which RBP expression increases in the endometrium of both cyclic and pregnant gilts on d 12 and 15; however, expression of RBP decreased by d 18 in the cyclic gilts (Harney et al. 1993). Additionally, the enzymes LRAT and ALDH1A1 as well as RBP4 were greatest on d 60 of pregnancy in the endometrium which may suggest RBP4 could be maintaining vitamin A homeostasis by facilitating its targeted delivery to the endometrium, then LRAT converts retinol into retinyl esters for vitamin A storage, while ALDH1A1 oxidizes retinal to retinoic acid. In the pig, gestational placental development is deemed complete in terms of weight, surface area, and numbers of areolae by approximately d 60 to 70 (Bazer and Johnson 2014; Almeida and Dias 2022). Moreover, areolae function are critical sites for histotrophic nutrition, facilitating the absorption of uterine gland–derived secretions rich in proteins, lipids, carbohydrates, uteroferrin, and retinol-binding proteins (Bazer and Johnson 2014). After this point in gestation, there is rapid fetal growth and increased uterine blood flow; therefore, the potential stored retinyl esters for vitamin A storage (via LRAT) and the retinoic acid (via ALDH1A1) in the endometrium may then be utilized for these purposes. In the swine industry, genetic selection for increased sow prolificacy (i.e. litter size) has likely limited the uterine and postnatal transfer of some micronutrients. Specifically, Matte and Audet (2020) identified three micronutrients including vitamins A and D, and copper could be limited for piglets during the postnatal period despite dietary supplementations to sows. Interestingly, it has been previously shown that an intramuscular injection of both vitamins A and D on d 85 of gestation to crossbred sows resulted in fewer stillborn piglets compared to a control injection of saline, suggesting improved offspring survival at birth (Yankocy et al. 2025). Future investigations should examine the utilization of vitamin A by various reproductive tissues and the developing fetus during mid- to late-gestation and possibly at farrowing and during prenatal development.

## Conclusion

Vitamin A is an essential nutrient involved in various biological processes observed across species, including reproduction. Investigations examining the role of vitamin A and metabolites in female reproduction have been an ongoing effort for many decades, where previous findings reveal its potential involvement in ovarian and CL steroidogenesis, among other reproductive functions. The current study aimed to quantify genes associated with vitamin A metabolism in the CL and endometrium and determine circulating plasma concentrations of P4 and vitamin A of d 15 cyclic or pregnant pigs throughout various points in gestation. Results of the current study suggest genes encoding enzymes involved in vitamin A metabolism (*ALDH1A1, ADH1C, RBP2*, and *RBP4*) are elevated in the CL during the period of early gestation when the anti-luteolytic mechanism of maternal recognition of pregnancy is occurring. Additionally, certain genes encoding enzymes involved in vitamin A metabolism (*ALDH1A1* and *LRAT*) are elevated mid- to late-gestation in the endometrium of pregnant pigs. Prospective investigations should continue to measure the expression of genes, as well as localize and quantify the expressions of proteins, such as enzymes and binding proteins, crucial to vitamin A metabolism and transport in reproductive tissues of cyclic and pregnant gilts to further support this exploration. Overall, more research, both examining baseline endogenous levels and supplementation effects of vitamin A, is required to clarify the reproductive mechanisms of vitamin A in the pig to mitigate pregnancy loss and improve reproductive throughput, which will ultimately benefit the swine industry.

## Supplementary Material

skag301_Supplementary_Data

## Abbreviations

ADH1C: alcohol dehydrogenase 1C
ALDH1A1: aldehyde dehydrogenase 1 family member A1
CV: coefficient of variation
CL: corpora lutea
IL1B2: interleukin 1beta 2
LRAT: lecithin retinol acyltransferase
P4: progesterone
RBP: retinol-binding protein
RDH12: retinol dehydrogenase 12
RIA: radioimmunoassay
RPL7: ribosomal protein L7
