# Supplementary material for: Changes in gene expression associated with vitamin A metabolism in the corpora lutea and endometria during d 15 of the estrous cycle and throughout gestation in gilts
Source: J Anim Sci. 2026 Sep 18;104:skag301. doi: 10.1093/jas/skag301 (PMC13656751; doi:10.1093/jas/skag301)
Supplement: Supplementary file 1 [file skag301_supplementary_data.docx]

**Supplementary Table 1.** Overall correlations between circulating vitamin A concentrations and relative mRNA expression of *ALDH1A1, ADH1C, LRAT, RBP2,* and *RBP4* in CL and endometrial tissue in gilts.

|  | Correlation coefficient (*r*) | *P-*value |
| --- | --- | --- |
| **Corpora lutea** |  |  |
| *ALDH1A1* | -0.125 | 0.462 |
| *ADH1C* | -0.233 | 0.172 |
| *LRAT* | 0.072 | 0.671 |
| *RBP2* | -0.035 | 0.838 |
| *RBP4* | -0.006 | 0.970 |
|  |  |  |
| **Endometrial** |  |  |
| *ALDH1A1* | 0.237 | 0.177 |
| *ADH1C* | -0.208 | 0.262 |
| *LRAT* | 0.232 | 0.195 |
| *RBP2* | 0.112 | 0.522 |
| *RBP4* | 0.299 | 0.086**ǂ** |

**ǂ** indicates trending towards significant at 0.05 < *P* ≤ 0.1

**Supplementary Figure 1**. Correlation between circulating vitamin A concentrations and relative mRNA expression of *RBP4* in endometrial tissue during day 15 of the estrous cycle (Cyclic) and throughout various timepoints of gestation (Days 11, 15, 60, and 90) in gilts.
